# Supplementary material for: An Amish founder population reveals rare-population genetic determinants of the human lipidome
Source: Commun Biol. 2022 Apr 7;5:334. doi: 10.1038/s42003-022-03291-2 (PMC8989972; doi:10.1038/s42003-022-03291-2)
Supplement: Supplementary file 2 — Supplementary Information [file 42003_2022_3291_MOESM2_ESM.pdf]

Supplementary Figures

Supplementary Figure 1: Heatmap for pairwise phenotype (upper triangle) and genetic (lower triangle) correlation between lipid species and traditional lipids. White squares in the heatmap represent genetic correlation estimates outside the interval [-1,1] that were set to missing. Such estimates are due to one or both traits having low heritability, thus, reducing power to estimate genetic correlation robustly.

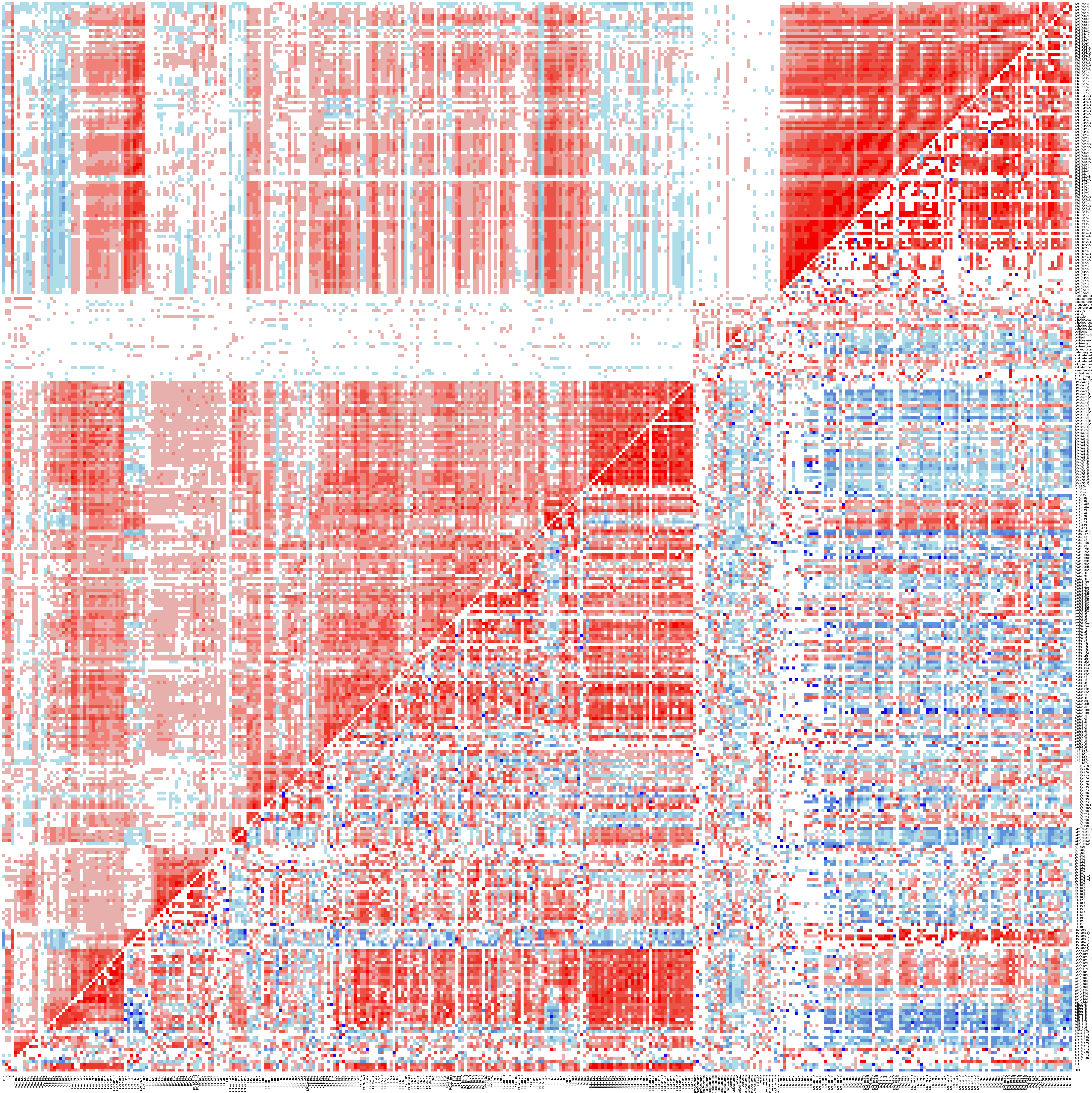

Supplementary Figure 2: Box plot comparing genetic and phenotypic correlations of all triacyclglycerol (TAG) species stratified by number of species (0,1 or 2) in the correlation containing 54 or more carbons and 4 or more double bonds. N is the number of pairs in the group. P-values for differences between each 2 groups were calculated by two sided Student's t-test. The upper, center, and lower line of the boxplot indicates third quartile (Q3), median, first quartile (Q1), respectively. The upper and lower whisker of the boxplot indicates Q3 +1.5 * interquartile range (IQR) and Q1 −1.5 * IQR. Outliers are suppressed from the plot for readability.

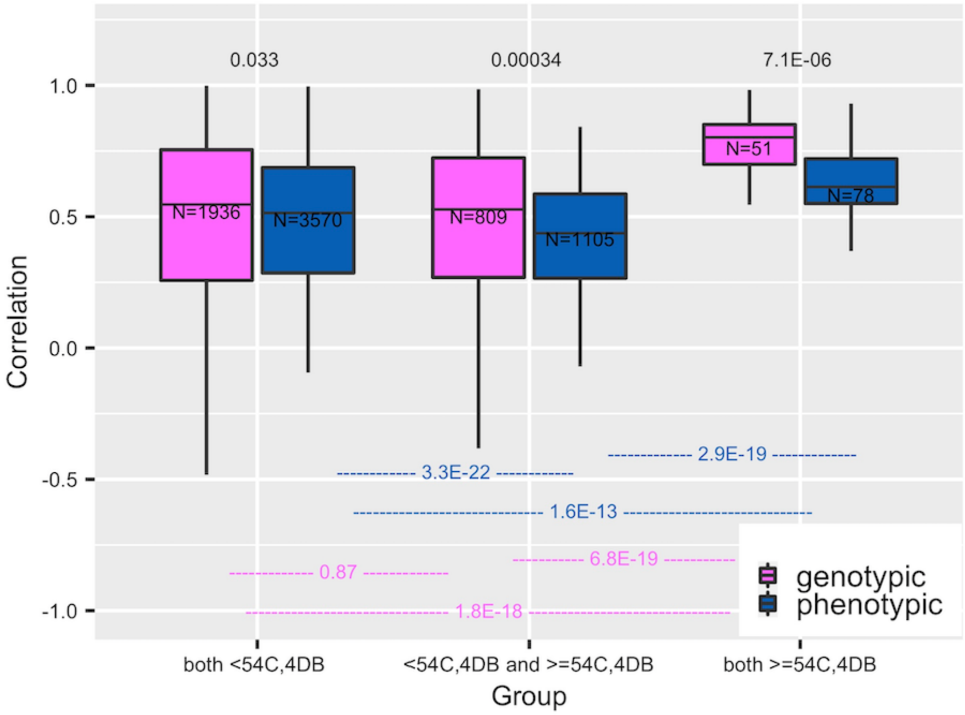

Supplementary Figure 3: Lipidome contribution to traditional lipids. The heritability (h^2) and the proportion of lipid class variance (Lipidomic class) estimated from a variance component model including lipid class species as a random effect. The exact estimates are in Supplementary Table 4. Abbreviations: ACT acylcarnitine, CE cholesteryl ester, Cer ceramide, DAG diglycerides, FA fatty acid, GlcCer glycosphingolipid, LPC lysophosphatidylcholine, LPE lysophosphatidylethanolamine, PC phosphatidylcholine, PE phosphatidylethanolamine, PI phosphatidylinositol, SM sphingomyelin, TAG triglyceride.

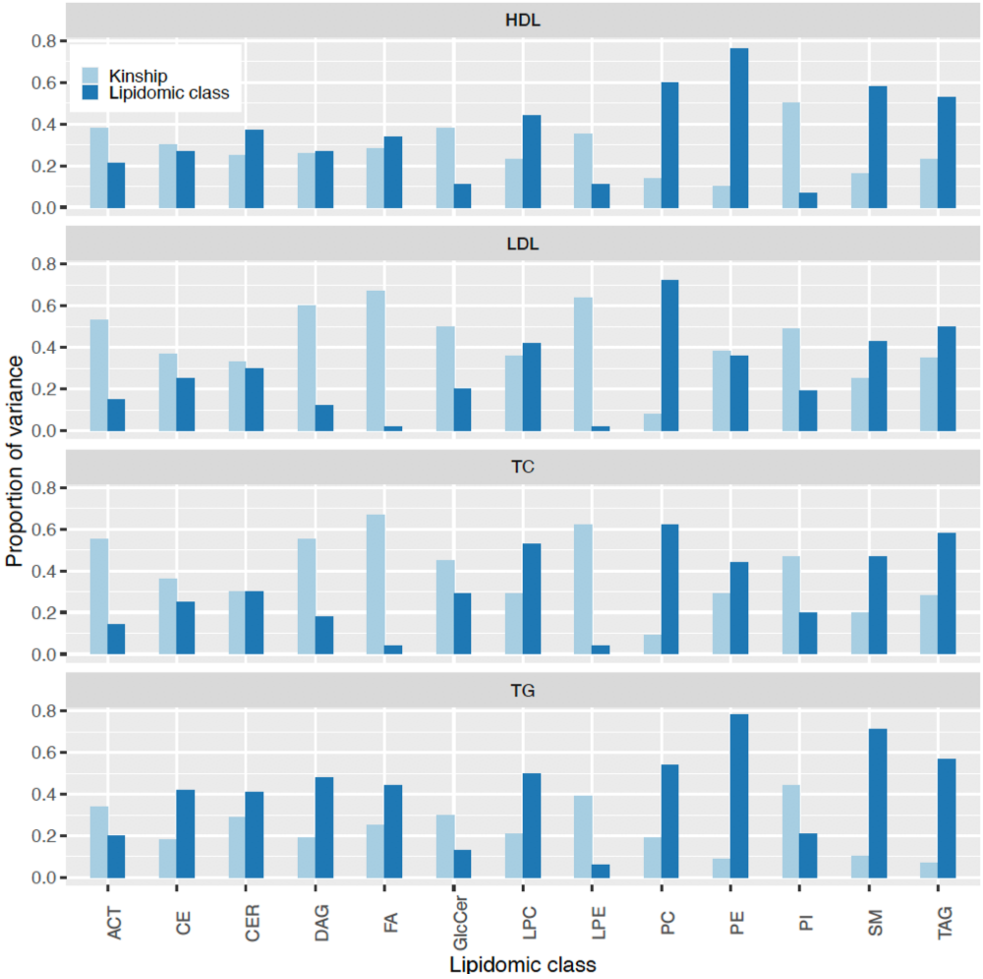
HDL
LDL
TC
TG
Kinship
Lipidomic class
Proportion of variance
0.0
0.2
0.4
0.6
0.8
ACT
CE
CER
DAG
FA
GlcCer
LPC
LPE
PC
PE
PI
SM
TAG
Lipidomic class

Supplementary Figure 4: The association of known lipid variants with lipid species (The top Manhattan plot) and traditional lipids (the bottom Manhattan plot)
Both the number and statistical significance is greater in the lipidomic plot, showing the higher power of lipidomics compared to traditional lipids to identify genetic associations. All p-values based on t-test using additive genetic model. Abbreviations: ACT acylcarnitine, CE cholesteryl ester, Cer ceramide, DG diglycerides, FA fatty acid, GlcCer glycosphingolipid, LPC lysophosphatidylcholine, LPE lysophosphatidylethanolamine, PC phosphatidylcholine, PE phosphatidylethanolamine, PI phosphatidylinositol, SM sphingomyelin, TAG triglyceride.

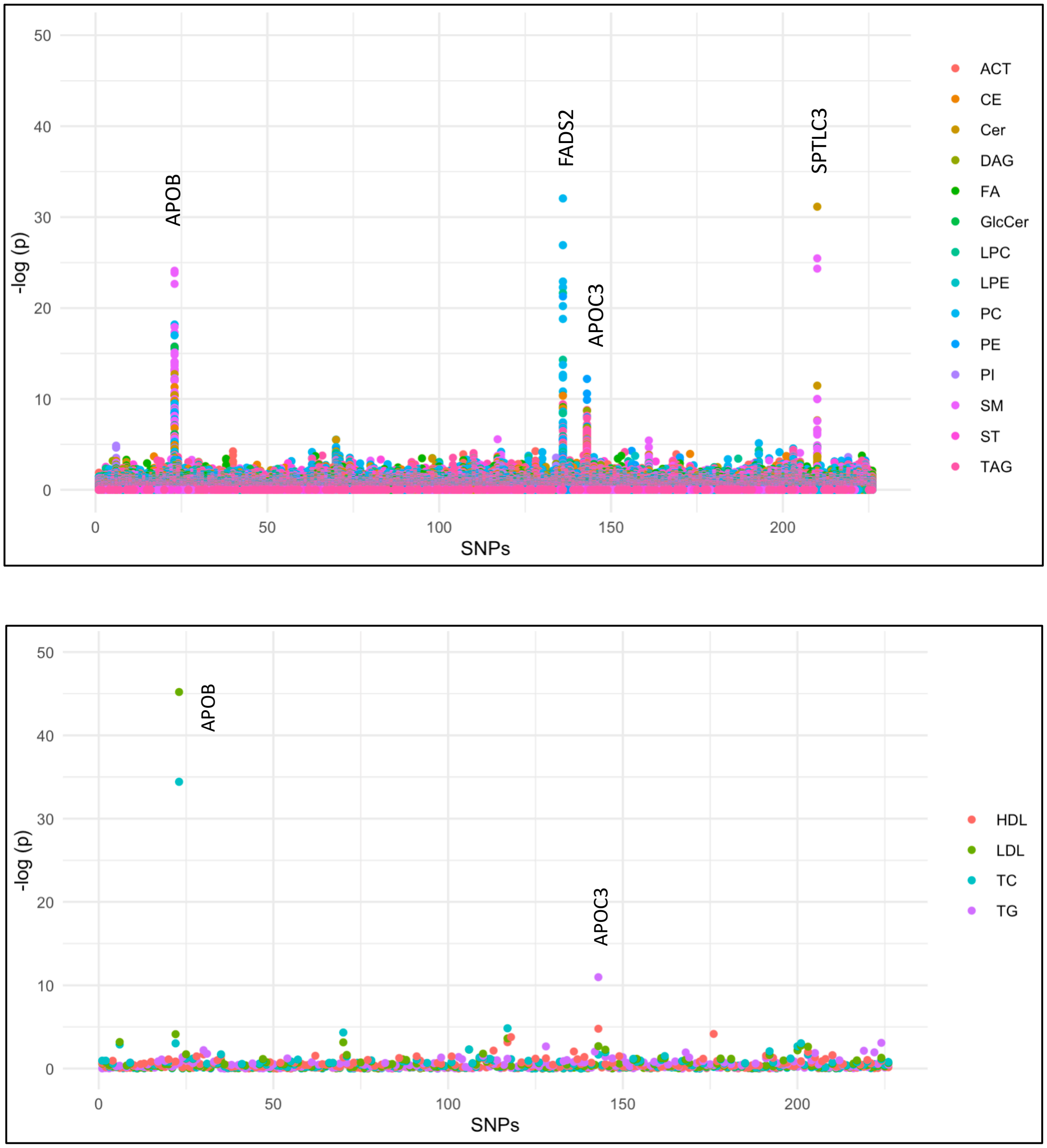

Supplementary Table 1: Functional annotation for the top 27 variants in chromosome 9 locus highlight the intronic variant (rs531892793, p=3.9E-17) as a strong potentially functional variant in this region. All p-values based on t-test using additive genetic model. r^2, D': r2 and D prime correlation with the top variant. RglmDB: RegulomeDB classification[1], eigenPC score[2], Dnase: DNase I hypersensitivity site[3], Reg: The ensembl regulatory build[4], and chromatin state in four different tissue[5] (islet, liver, adipose, and skeletal muscle[5]). 5.Tx (Strong_transcription), 6.TxWk (Weak_transcription), 8.EnhG (Genic_Enhancer1), 9.EnhA1 (Active_Enhancer1), 10.EnhA2 (Active_Enhancer1), 11.EnhWk (Weak_Enhancer), 16.RPC (Repressed_PolyComb), 17.RPCWk (Weak_Repressed_PolyComb), 18.Quies (Quiescent/low).

rsNum	pVal_SNP	r^2	D'	RglmDB	eigenPC	Dnase	Reg	islets	liver	adipose	sklmus
rs7863920	6.22E-18	1	1	7	-0.257			18.Quies	17.RPCWk	18.Quies	18.Quies
rs146764160	9.37E-18	0.79	0.94	6	-0.251			18.Quies	18.Quies	6.TxWk	18.Quies
rs138715201	9.37E-18	0.79	0.94	6	-0.158			6.TxWk	6.TxWk	6.TxWk	6.TxWk
rs140093477	9.37E-18	0.79	0.94	4	0.105	371	open	16.RPC	18.Quies	16.RPC	16.RPC
rs542505754	9.37E-18	0.79	0.94	6	-0.139			5.Tx	5.Tx	8.EnhG	5.Tx
rs186979747	9.37E-18	0.79	0.94	7	-0.136			6.TxWk	6.TxWk	11.EnhWk	6.TxWk
rs952789069	1.10E-17	0.86	0.98	7	-0.094			17.RPCWk	18.Quies	17.RPCWk	17.RPCWk
rs144332784	1.24E-17	0.86	0.98	7	-0.218			17.RPCWk	17.RPCWk	17.RPCWk	18.Quies
rs190844068	1.24E-17	0.86	0.98	6	-0.168			17.RPCWk	17.RPCWk	18.Quies	17.RPCWk
rs185511591	1.65E-17	0.86	0.98	7	-0.198			18.Quies	18.Quies	18.Quies	18.Quies
rs566604550	1.65E-17	0.86	0.98	6	-0.284			18.Quies	18.Quies	18.Quies	18.Quies
rs974137970	1.69E-17	0.86	0.98	7	-0.22			17.RPCWk	17.RPCWk	18.Quies	18.Quies
rs181234756	1.69E-17	0.86	0.99	5	-0.177			17.RPCWk	6.TxWk	18.Quies	18.Quies
rs927992688	1.69E-17	0.86	0.98	6	-0.152			18.Quies	18.Quies	18.Quies	18.Quies
rs146981196	1.69E-17	0.86	0.98	6	-0.012			18.Quies	17.RPCWk	18.Quies	18.Quies
rs141683313	1.71E-17	0.86	0.95	6	-0.108			18.Quies	6.TxWk	6.TxWk	6.TxWk
rs1014518150	1.76E-17	0.86	0.95	6	-0.173			17.RPCWk	17.RPCWk	6.TxWk	18.Quies
rs533456288	3.83E-17	0.78	0.93	5	-0.175	214		6.TxWk	18.Quies	18.Quies	17.RPCWk
rs543685197	3.86E-17	0.78	0.93	5	-0.039	313		6.TxWk	18.Quies	18.Quies	17.RPCWk
rs531892793	4.00E-17	0.78	0.93	2a	3.501	1000	proFR	6.TxWk	8.EnhG	9.EnhA1	6.TxWk
rs956743578	4.12E-17	0.78	0.93	5	-0.009			6.TxWk	5.Tx	6.TxWk	6.TxWk
rs528148931	4.15E-17	0.78	0.93	5	-0.14			10.EnhA2	9.EnhA1	6.TxWk	11.EnhWk
rs993853922	4.15E-17	0.78	0.93	5	0.018			6.TxWk	5.Tx	6.TxWk	6.TxWk
rs978182891	4.23E-17	0.86	0.98	7	-0.211			17.RPCWk	16.RPC	18.Quies	18.Quies
rs554319484	4.23E-17	0.86	0.98	7	-0.209			17.RPCWk	16.RPC	18.Quies	18.Quies
rs1011013571	1.36E-16	0.86	0.95	7	-0.145			17.RPCWk	17.RPCWk	6.TxWk	6.TxWk
rs78967418	8.59E-16	0.76	0.87	6	-0.205			17.RPCWk	17.RPCWk	6.TxWk	18.Quies

References:

1. Boyle, A.P., et al., Annotation of functional variation in personal genomes using RegulomeDB. *Genome research*, 2012. 22(9): p. 1790-1797.

2. Ionita-Laza, I., et al., A spectral approach integrating functional genomic annotations for coding and noncoding variants. *Nat Genet*, 2016. 48(2): p. 214-20.

3. Snyder, M.P., et al., Perspectives on ENCODE. *Nature*, 2020. 583(7818): p. 693-698.

4. Zerbino, D.R., et al., The ensembl regulatory build. *Genome Biol*, 2015. 16: p. 56.

5. Varshney, A., et al., Genetic regulatory signatures underlying islet gene expression and type 2 diabetes. *Proc Natl Acad Sci U S A*, 2017. 114(9): p. 2301-2306.
