# Supplementary material for: An Amish founder population reveals rare-population genetic determinants of the human lipidome
Source: Commun Biol. 2022 Apr 7;5:334. doi: 10.1038/s42003-022-03291-2 (PMC8989972; doi:10.1038/s42003-022-03291-2)
Supplement: Supplementary file 3 — Description of Additional Supplementary Files [file 42003_2022_3291_MOESM3_ESM.pdf]

Description of Additional Supplementary Files

Supplementary Data 1:

Demographic and clinical characteristics (mean (sd)) of the HAPI discovery cohort and the GOLDN replication cohort.

Supplementary Data 2:

Heritability estimates and genomic inflation factor of the 355 lipidomic species tested in the discovery GWAS.

Supplementary Data 3:

Pairwise phenotype (upper triangle) and genetic (lower triangle) correlation between lipid species and traditional lipids. Blank genetic correlations indicate the maximum likelihood estimates were outside valid correlation bounds.

Supplementary Data 4:

Single lipidome contribution to traditional lipids. Results from joint estimate of heritability and lipidomic class variance for each traditional lipid.

Supplementary Data 5:

Cumulative lipidome contribution to traditional lipids. Results from forward sequential variance component model was run starting with heritability, then at each step the LRT p-value of the current model vs. the current model with a remaining lipid class was computed. The lipid class with lowest p-value was then added to the current model. The process continued until the best LRT p-value > 0.95.

Supplementary Data 6:

All GWAS results with p-value <5.0E-08 in the Amish and GOLDN replication association results.

Supplementary Data 7:

Results of the significant (p<4.5E-10) 5 Amish enriched loci in the top associated trait and GOLDN replication association results for the same trait and variant.

Supplementary Data 8:

Amish association results for Tabassum results.

Supplementary Data 9:

Association results of the 1602 top lipidomic associated variants with four traditional lipids HDL, LDL, TC and TG.
