# Supplementary material for: An Amish founder population reveals rare-population genetic determinants of the human lipidome
Source: Commun Biol. 2022 Apr 7;5:334. doi: 10.1038/s42003-022-03291-2 (PMC8989972; doi:10.1038/s42003-022-03291-2)
Supplement: Supplementary file 13 — Reporting Summary [file 42003_2022_3291_MOESM13_ESM.pdf]

nature portfolio

Corresponding author(s): May Montasser

Last updated by author(s): Feb 22, 2022

Reporting Summary

Nature Portfolio wishes to improve the reproducibility of the work that we publish. This form provides structure for consistency and transparency in reporting. For further information on Nature Portfolio policies, see our Editorial Policies and the Editorial Policy Checklist.

Statistics

For all statistical analyses, confirm that the following items are present in the figure legend, table legend, main text, or Methods section.

n/a	Confirmed	
☐	☒	The exact sample size (*n*) for each experimental group/condition, given as a discrete number and unit of measurement
☐	☒	A statement on whether measurements were taken from distinct samples or whether the same sample was measured repeatedly
☐	☒	The statistical test(s) used AND whether they are one- or two-sided<br>*Only common tests should be described solely by name; describe more complex techniques in the Methods section.*
☐	☒	A description of all covariates tested
☐	☒	A description of any assumptions or corrections, such as tests of normality and adjustment for multiple comparisons
☐	☒	A full description of the statistical parameters including central tendency (e.g. means) or other basic estimates (e.g. regression coefficient) AND variation (e.g. standard deviation) or associated estimates of uncertainty (e.g. confidence intervals)
☐	☒	For null hypothesis testing, the test statistic (e.g. *F*, *t*, *r*) with confidence intervals, effect sizes, degrees of freedom and *P* value noted<br>*Give P values as exact values whenever suitable.*
☒	☐	For Bayesian analysis, information on the choice of priors and Markov chain Monte Carlo settings
☒	☐	For hierarchical and complex designs, identification of the appropriate level for tests and full reporting of outcomes
☐	☒	Estimates of effect sizes (e.g. Cohen's *d*, Pearson's *r*), indicating how they were calculated

Our web collection on statistics for biologists contains articles on many of the points above.

Software and code

Policy information about availability of computer code

Data collection	*Provide a description of all commercial, open source and custom code used to collect the data in this study, specifying the version used OR state that no software was used.*
Data analysis	MMAP https://github.com/MMAP<br>ENCORE https://encore.sph.umich.edu

For manuscripts utilizing custom algorithms or software that are central to the research but not yet described in published literature, software must be made available to editors and reviewers. We strongly encourage code deposition in a community repository (e.g. GitHub). See the Nature Portfolio guidelines for submitting code & software for further information.

Data

Policy information about availability of data

All manuscripts must include a data availability statement. This statement should provide the following information, where applicable:

- Accession codes, unique identifiers, or web links for publicly available datasets
- A description of any restrictions on data availability
- For clinical datasets or third party data, please ensure that the statement adheres to our policy

Amish phenotype, genotype, and lipidome data are available to academic investigator via a Data Use Agreement with UMB. GOLDN phenotype, genotype, and lipidome data are available to academic investigator via a Data Use Agreement with UAB

Field-specific reporting

Please select the one below that is the best fit for your research. If you are not sure, read the appropriate sections before making your selection.

☒ Life sciences ☐ Behavioural & social sciences ☐ Ecological, evolutionary & environmental sciences

For a reference copy of the document with all sections, see nature.com/documents/nr-reporting-summary-flat.pdf

Life sciences study design

All studies must disclose on these points even when the disclosure is negative.

Sample size	No power calculation was needed in advance and we used all samples available.
Data exclusions	All samples with phenotype, genotypes and lipidome data were used.
Replication	GOLDN study was used for replication
Randomization	N/A
Blinding	N/A

Reporting for specific materials, systems and methods

We require information from authors about some types of materials, experimental systems and methods used in many studies. Here, indicate whether each material, system or method listed is relevant to your study. If you are not sure if a list item applies to your research, read the appropriate section before selecting a response.

Materials & experimental systems

n/a	Involved in the study	
☒	☐	Antibodies
☒	☐	Eukaryotic cell lines
☒	☐	Palaeontology and archaeology
☒	☐	Animals and other organisms
☐	☒	Human research participants
☒	☐	Clinical data
☒	☐	Dual use research of concern

Methods

n/a	Involved in the study	
☒	☐	ChIP-seq
☒	☐	Flow cytometry
☒	☐	MRI-based neuroimaging

Human research participants

Policy information about studies involving human research participants

Population characteristics	The Old Order Amish (OOA) population of Lancaster County, PA immigrated to the Colonies from Western Europe in the early 1700's. There are now about 40,000 OOA individuals in the Lancaster area, nearly all of whom can trace their ancestry back 12-14 generations to approximately 750 founders. Participants in this study were middle age on average with balanced representation of males and females, and BMI of about 26. The LDL-C levels is borderline high due to the enrichment of the APOB p.Arg3527Gln familial hypercholestrolimia variant, and good profile of HDL-C and TG due to the enrichment of the APOC3 p.Arg19Ter cardoprotective variant.<br><br>GOLDN (Genetics of Lipid Lowering Drugs and Diet Network), the largest study of postprandial dyslipidemia that offers NMR, clinical lipid, and lipidomic measures, was initiated to assess the interaction of genetic factors with environmental interventions (intake of a high-fat meal and/or fenofibrate treatment). Participants in this study were middle age on average with balanced representation of males and females, and BMI of about 28, and normal lipid profile.
Recruitment	HAPI was initiated in 2002 to identify the genetic and environmental determinants of responses (blood pressure, triglyceride excursion and platelet aggregation) to four short-term interventions including a cold pressor stress test, a high salt diet, a high fat challenge, and an aspirin therapy in a four-week time period. HAPI recruited 1,003 OOA, and the interventions were carried out in 868 relatively healthy OOA adults (>= 20 years of age). Participants were asked to discontinue the use of all medications, vitamins and supplements for at least 7 days prior to the first visit and during the interventions, to fast at least 12 hours prior to their visit, and to restrain themselves from doing excessive physical activity on the morning of their appointment. Baseline blood drawn from 650 participants was used for the lipidomic profiling in this study. The study protocol was approved by the institutional review board at the University of Maryland. Informed consent was obtained from each of the study participants.<br><br>GOLDN recruited European American families with at least two siblings from two field centers (Minneapolis, MN and Salt

Lake City, UT) of the Family Heart Study (FHS). Participants were excluded if they 1) had fasting triglycerides (TGs) ≥ 1500 mg/dL, 2) had a history of kidney, liver, pancreas, or gallbladder disease, recent myocardial infarction or revascularization, or nutrient malabsorption, 3) reported a current use of insulin, and 4) were pregnant or lactating.

Ethics oversight	The institutional review boards at the University of Maryland and the University of Alabama

Note that full information on the approval of the study protocol must also be provided in the manuscript.
